# Electroacupuncture Improves Baroreflex and *γ*-Aminobutyric Acid Type B Receptor-Mediated Responses in the Nucleus Tractus Solitarii of Hypertensive Rats

**DOI:** 10.1155/2018/8919347

**Published:** 2018-09-30

**Authors:** Qi Zhang, Ying-Ying Tan, Xiao-hua Liu, Fan-Rong Yao, Dong-Yuan Cao

**Affiliations:** ^1^Shaanxi Key Laboratory of Chinese Medicine Encephalopathy, Shaanxi University of Chinese Medicine, Xianyang, Shaanxi 712046, China; ^2^Department of Pharmacology & Toxicology, Brody School of Medicine, East Carolina University, Greenville, NC 27834, USA; ^3^Key Laboratory of Shaanxi Province for Craniofacial Precision Medicine Research, Research Center of Stomatology, Xi'an Jiaotong University College of Stomatology, Xi'an, Shaanxi 710004, China

## Abstract

Electroacupuncture (EA) has been reported to benefit hypertension, but the underlying mechanisms are still unclear. We hypothesized that EA attenuates hypertension, in part, through modulation of *γ*-aminobutyric acid (GABA) receptor function in the nucleus tractus solitarii (NTS). In the present study, the long-term effect of EA on GABA receptor function and expression was examined in the NTS of two-kidney, one-clip (2K1C) renovascular hypertensive rats. EA (0.1–0.4 mA, 2 and 15 Hz) was applied at Zusanli (ST36) acupoints overlying the deep fibular nerve for 30 min once a day for two weeks. The results showed that long-term EA treatment improved blood pressure (BP) and markedly restored the baroreflex response in 2K1C hypertensive rats. The increased pressor and depressor responses to microinjection of GABA_B_ receptor agonist and antagonist into the NTS in the hypertensive rats were blunted by the EA treatment. Moreover, EA treatment attenuated the increased GABA_B_ receptor expression in the NTS of hypertensive rats. In contrast, EA had no significant effect on the GABA_A_ receptor function and expression in the NTS of 2K1C hypertensive rats. These findings suggest that the beneficial effects of EA on renovascular hypertension may be through modulation of functional GABA_B_ receptors in the NTS.

## 1. Introduction

Acupuncture has been used for centuries in treatment of various disorders, including cardiovascular disease (for review, see [1, 2]). Electroacupuncture (EA) is a more effective way of administering acupuncture, which applies a pulsating electrical current to acupuncture needles for acupoint stimulation. Clinical and experimental studies indicate that low-frequency EA at Zusanli (ST36) acupoint may have therapeutic and modulatory effects on some types of hypertension [1, 3–5]. It has been demonstrated that the nervous system, neurotransmitters, and endogenous substances are involved in EA treatment [6–8]. However, the underlying mechanisms for beneficial antihypertensive responses of EA are unclear.

It is well known that elevated sympathetic outflow and impaired baroreflex function contribute to the development of hypertension [9]. The evidence suggests that EA could lower sympathetic activity and significantly inhibit the sympathoexcitatory reflex responses in rats [10–12]. It has also been reported that sensory stimulation of the hindlimb somatic afferent modifies neuronal activity of the nucleus tractus solitarii (NTS) [13]. The NTS is the main integration center for regulating autonomic reflex and sympathetic outflow [14]. Thus, neuronal activity in the NTS is one of the important targets of EA for modulating sympathoexcitatory reflex function.

Within the NTS, GABAergic inhibition plays an important role in baroreflex signal processing. There is considerable evidence suggesting increased GABAergic inhibition in the NTS contributes to the development of hypertension [14–16]. Moreover, the NTS contains a high density of both GABA_B_ and GABA_A_ receptors [17]. GABA_B_ receptors are metabotropic G protein-coupled receptors that mediate presynaptic and postsynaptic inhibitions by reductions in calcium conductance or increases in potassium conductance. Our previous studies and others showed that hypertensive rats exhibit enhanced GABA_B_ receptor function and regulation within the NTS [18–21]. Thus, manipulations resulting in changes in GABA_B_ receptor function in the NTS may have a greater effect on blood pressure (BP) regulation in hypertension. However, whether alterations in GABA_B_ function contribute to the antihypertensive effect of EA is unknown.

In the present study, we tested the hypothesis that EA reduced BP and improved baroreflex response in renovascular hypertensive rats, as well as the beneficial antihypertensive effect of EA was associated with modulation of functional GABA_B_ receptors within the NTS of hypertensive rats.

## 2. Methods

### 2.1. Animals

Adult male Sprague-Dawley rats, weighing 200–210 g, were used in this study. All animals were provided by the Laboratory Animal Center of Xi'an Jiaotong University and housed under controlled conditions with a 12 : 12-h light-dark cycle. Food and water were available to the animals ad libitum. All protocols were approved by the Institutional Animal Care and Use Committee of Shaanxi University of Chinese Medicine.

### 2.2. Blood Pressure Measurement

Conscious BP recording was carried out with a radiotelemetry system, as described previously [21]. Ten days before making a two-kidney, one-clip (2K1C) model or sham operation, the rats were anesthetized with isoflurane (3%), which was delivered through a nose cone. A telemetry BP probe (model TA11PA-C40, Data Sciences International, St. Paul, MN, USA) was positioned intra-abdominally and secured to the ventral abdominal muscle with the catheter inserted into the lower abdominal aorta. The telemetry signals received by the device were processed and digitized as radiofrequency data, which were recorded and stored in a computer using the Dataquest IV system (Data Sciences International, St. Paul, MN, USA), and the mean values of BP and heart rate (HR) were calculated in a conscious state. Continuous recordings were started 4 days after the probe implantation.

For the microinjection studies, acute BP recording was performed using PE50 catheters under isoflurane (3%) anesthesia. PE50 catheters filled with heparinized saline (100 IU/mL) were placed in the right femoral artery and connected to a BP transducer and a bridge amplifier (AD Instrument, Bella Vista, Australia). The BP and HR data were collected and analyzed with PowerLab software (AD Instrument, Bella Vista, Australia).

### 2.3. Experimental Group

Male rats were randomly divided into four groups after one week of adaptation: the sham group, 2K1C hypertensive group, 2K1C plus EA treatment group (2K1C + EA), and 2K1C plus sham EA treatment group (2K1C + SEA). The 2K1C hypertensive model was established as previously described [22]. Briefly, after basal BP recording for a 3-day control period using radiotelemetry, the rats were anesthetized with pentobarbital (i.p., 40 mg/kg). The left kidney was accessed through a left lateral incision and partly obstructed with a silver clip with an internal diameter of 0.20 mm. Sham-operated rats were performed the same surgical procedures without clip placement.

### 2.4. EA Application

Fourteen days after 2K1C or sham surgery, the EA application was performed as previously described [5, 11]. Rats were adapted and handled gently for 30 min each day, for 3 days before the beginning of the experiment. BP was recorded using radiotelemetry each day before EA treatment. Stainless steel needles (0.16 mm diameter) were inserted bilaterally in the ST36 acupoint, which was located in the anterolateral portion of the hindlimb, in the middle of the cranial tibial muscle, 5 mm below the capitulum fibulae, 7 mm deep from the skin surface, and is innervated by the deep fibular nerve [23]. As the sham EA control group, a nonacupuncture point located at the junction between the tail and buttock was applied stimulation by the needle just inserting into the epidermis of the skin. The electrical stimulation was performed using Hans-200A electrostimulator (Beijing Sheng Da Medical Instrument Center, Beijing, China) at alternate frequencies (2 Hz and 15 Hz), with 0.5 ms in duration and certain intensity (≤4 mA) which elicited slight muscle contraction or movement of the paw. Either EA or SEA stimulation was applied for 30 min once a day for 2 weeks. In the sham control group and 2K1C group, rats were stayed for a 30-minute period with needle insertion but without electrical stimulation of ST36 acupoints. All procedures were performed by the same researcher.

### 2.5. Baroreflex Responses

At the end of the EA treatment, rats were anesthetized with inhalation of isoflurane (3%). PE50 catheters filled with heparinized saline (100 IU/mL) were placed in the right femoral artery for BP monitoring, and the ipsilateral femoral vein was inserted with the PE10 venous cannula for intravenous administration. Following the baseline BP and HR recordings, baroreflex sensitivity was evaluated by administration of phenylephrine (5 *μ*g kg^−1^, i.v.) and sodium nitroprusside (30 *μ*g kg^−1^, i.v.) by intravenous bolus injections to induce either an increase or decrease in BP, respectively, and subsequent bradycardic or tachycardic responses. A 10 min interval between injections was necessary for blood pressure to return to baseline. The one second mean HR values in response to 10 mmHg incremental changes in MAP (from 5 mmHg to 35 mmHg) were analyzed. The values were plotted; the differences between groups were calculated as described previously [24].

Spontaneous baroreflex sensitivity was assessed by the sequence method using the radiotelemetry data as previously described [25–27]. Analysis was performed with HemoLab Software Ver. 20.5. Baroreflex sequence was identified as the correlation coefficient (*r*) between systolic blood pressure and pulse interval values greater than or equal to 0.8. Baroreflex sensitivity was calculated from the slope of the linear regression lines between systolic blood pressure and the pulse interval of each baroreflex sequence, and the up sequence gain (in ms/mmHg), down sequence gain (in ms/mmHg), and overall baroreflex gain (in ms/mmHg) were determined.

### 2.6. Measurement of Urinary Norepinephrine Excretion

Urinary norepinephrine (NE) content was measured at 12 days after EA treatment. Urinary samples were collected after rats were placed in metabolism cages for 24 h, and NE enzyme immunoassay kit (Labor Diagnostika Nord KG, Nordhorn, Germany) was used to detect NE content in 24 h urinary excretion, as described previously. The sensitivity of this kit is 1.5 pg/sample.

### 2.7. Microinjection Experiments

At the end of the 14-day EA treatment, the BP and HR data were collected and analyzed with PowerLab software (AD Instrument, Bella Vista, Australia). GABA_B_ receptor agonist or antagonist was microinjected bilaterally into the NTS according to the procedures described previously [21]. In brief, the anesthetized animal was placed in a stereotaxic frame. After surgical exposure of the dorsal medulla oblongata, a multiple-barrel glass injection pipette (tip size 20–40 *μ*m) was positioned in the NTS. The coordinates for the NTS were determined from the Paxinos and Watson rat atlas, which is 0.5 mm rostral to the caudal tip of the area postrema, 0.5 mm lateral to the midline, and 0.5 mm below the dorsal surface. Proper placement was confirmed by checking for an L-glutamate-induced (300 pmol, in 50 nL) depressor response, which induced a characteristically abrupt decrease in BP (ΔBP > 35 mmHg) and HR (ΔHR > 30 beats/min) if the needle tip was located precisely in the NTS. After a responsive site was identified by L-glutamate, the probe remained in this site throughout the remainder of the experiment. The volume of microinjection was determined by the displacement of fluid meniscus in the micropipette barrel under a microscope. The GABA_B_ receptor antagonist (CGP-35348, 100 pmol in 50 nL, Sigma-Aldrich), GABA_B_ receptor agonist (baclofen, 50 pmol in 50 nL, Sigma-Aldrich), GABA_A_ receptor antagonist (bicuculline, 10 pmol in 50 nL, Sigma-Aldrich), or GABA_A_ receptor agonist (muscimol, 100 pmol in 50 nL, Sigma-Aldrich) was dissolved in saline and microinjected bilaterally into the NTS. Each dose for microinjection study was determined by preliminary experiments and our previous studies [18, 21]. The duration of each injection was 15 s. For bilateral microinjections of a given drug, the time interval between the two microinjections was <1 min. Rat body temperature was maintained in the range of 36.5–37.5°C with a heating pad. After the protocol, injection sites were marked with methylene blue dye (50 nL) and verified histologically.

### 2.8. Real-Time RT-PCR

The animals were killed with an excessive dose of sodium pentobarbital, and the NTS tissues were obtained using the micropunch technique as described previously [21]. In brief, using a rodent brain slicer (RuiWoDe Inc., RWD68709), 1 mm thick sections were obtained from the NTS caudal to calamus. The coordinates for the NTS were approximately −13.6 to −14.6 from the bregma, 0.5 mm lateral to the midline, and 0.5 mm below the dorsal surface of the brain stem. The NTS samples were identified and collected bilaterally from the section under a microscope by a punch made of 20-gauge stainless steel tubing. Real-time PCR was used to detect changes in the GABA_B_ and GABA_A_ receptor mRNA as detailed by us previously [18]. The total RNA was isolated from NTS tissue lysates using an RNeasy kit (Qiagen, Valencia, CA, USA) according to the manufacturer's instructions. TaqMan PCR probes for rat GABA_B_ (Rn00578911_m1), GABA_A_ (Rn01464079_m1), and 18 S rRNA (Rn03928990_g1) were obtained from Thermo Fisher Scientific (Carlsbad, CA, USA). Amplification was performed in an Applied Biosystems PRISM 7000 sequence detection system. A comparative cycle of threshold (CT) fluorescence method was applied with 18 S rRNA as the internal control. The final data of real-time PCR were presented as the ratio of the mRNA of interest to 18 S rRNA.

### 2.9. Western Blotting

The procedures were as described previously [21]. NTS samples were homogenized in an ice-cold lysing buffer containing 20 mM Tris·HCl (pH 6.8), 150 mM NaCl, 10% glycerol, 1% NP-40, and 8 *μ*L/mL inhibitor cocktail (125 mM PMSF, 2.5 mg/mL aprotinin, 2.5 mg/mL leupeptin, 2.5 mg/mL antipain, and 2.5 mg/mL chymostatin). The homogenate was centrifuged, and the supernatant was collected. The protein concentration was measured using a protein assay kit (Bio-Rad Laboratories, Hercules, CA, USA). The samples were boiled for 5 minutes, followed by loading on the SDS-PAGE gel (10 *μ*g of protein, 20 *μ*L per well) for electrophoresis using a Bio-Rad minigel apparatus at 100 V for 60 minutes. The fractionized protein on the gel was electrophoretically transferred onto the nitrocellulose membranes at 350 mA for 90 minutes. After blocking for 1 hour with 5% milk in Tris-buffered saline at room temperature, the membrane was probed with primary antibody (GABA_B_ rabbit polyclonal antibody, #AB5850, Chemicon, 1 : 1000; or GABA_A_ receptor rabbit polyclonal antibody, #AB5954, Chemicon, 1 : 500) and secondary antibody (goat anti-rabbit IgG horseradish peroxidase, Bio-Rad, 1 : 3000). Then the membrane was treated with enhanced chemiluminescence substrate (ECL Western blotting detection kit, Amersham Pharmacia Biotechnology, Piscataway, NJ, USA) for 5 minutes at room temperature. The specificity of GBR and GAR was tested by preincubation of the antibodies with the blocking peptides for 2 h at room temperature. The subsequent incubation steps were carried out as usual. The bands in the film were visualized and analyzed using Quantity One Software (Bio-Rad).

### 2.10. Statistical Analyses

All data are expressed as means ± SE. Comparisons between experimental groups were performed with ANOVA followed by a Newman-Keuls test. Differences were considered significant at *P* < 0.05.

## 3. Results

### 3.1. Effect of EA Treatment on the Development of 2K1C Hypertension and Baroreflex Response

**Figure 1:**
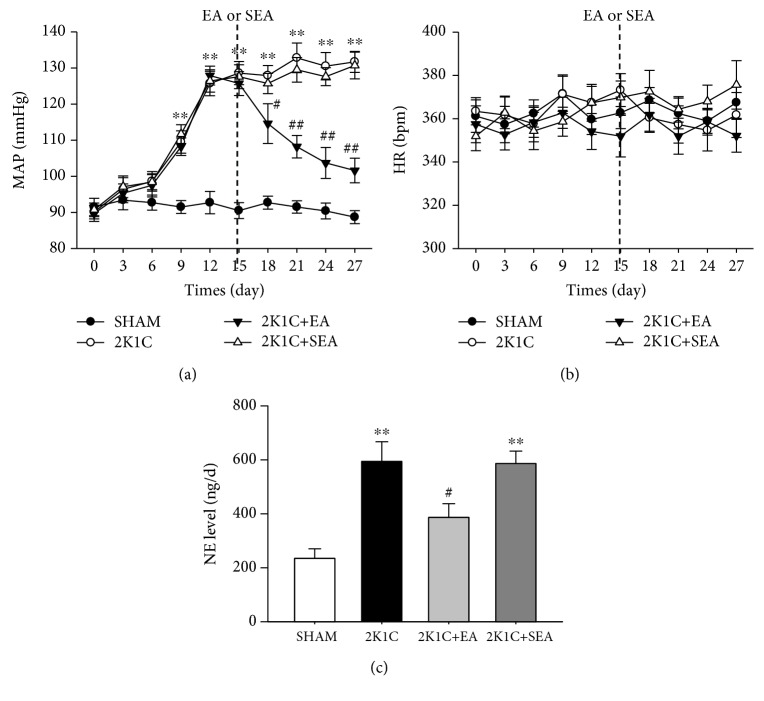
Long-term EA treatment attenuates hypertension in 2K1C hypertensive rats. (a) Time course of mean arterial pressure (MAP) in each group over 27 days. (b) Time course of heart rate (HR) in each group over 27 days. MAP and HR were measured by radiotelemetry. (c) The change in the urinary norepinephrine excretion at day 27 after the 2K1C hypertensive model was established. Data are presented as mean ± SE; *n* = 7–9 rats; ^∗^*P* < 0.05, ^∗∗^*P* < 0.01 vs. the sham group; ^#^*P* < 0.05, ^##^*P* < 0.01 vs. 2K1C group.

Mean arterial BP (MAP) and HR were measured via radiotelemetry. The baseline MAP and HR values were similar between the sham, 2K1C, 2K1C + EA, and 2K1C + SEA groups. As shown in Figure 1(a), the MAP was increased in the 2K1C group compared with that in the sham group. After 14 days of EA treatment, the 2K1C + EA group showed reduced MAP compared with the 2K1C group. However, the 2K1C + SEA group maintained high MAP compared with the sham group. In addition, no significant alterations in HR were observed in all groups (Figure 1(b)).

To determine whether chronic EA treatment altered sympathetic activities, urinary NE excretion was evaluated. As shown in Figure 1(c), urinary NE excretion markedly increased in the 2K1C group at day 27 after the 2K1C hypertensive model was established, and EA treatment significantly lowered the NE excretion in the 2K1C + EA group.

**Figure 2:**
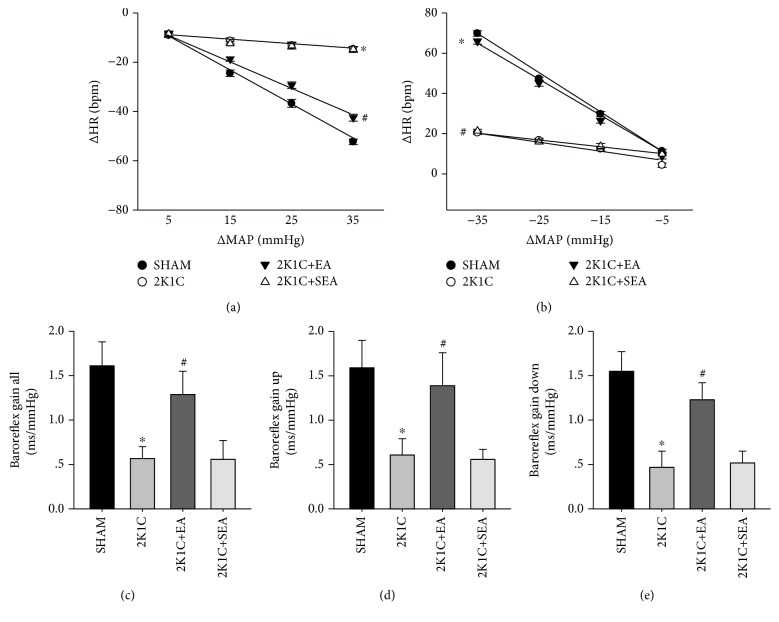
Long-term EA treatment restores the baroreflex response in 2K1C hypertensive rats. (a) Grouped heart rate (HR) baroreflex response to each 10 mmHg change in mean arterial pressure (MAP) elicited by phenylephrine. (b) Grouped heart rate (HR) baroreflex response to each 10 mmHg change in mean arterial pressure (MAP) evoked by sodium nitroprusside. (c–e) Spontaneous baroreflex analysis with overall baroreflex gain (combination of up and down sequences), up sequence gain, and down sequence gain (in ms/mmHg). Data are presented as mean ± SE; *n* = 7–9 rats; ^∗^*P* < 0.05 vs. the sham group; ^#^*P* < 0.05 vs. the 2K1C group.

The 2K1C group presented a reduction in baroreflex gain after administration of phenylephrine and sodium nitroprusside compared with the sham group, and the 2K1C + EA group exhibited the restored baroreflex sensitivity compared with the 2K1C group. However, the 2K1C + SEA group maintained the depressed baroreflex sensitivity compared with the sham group (Figures 2(a) and 2(b)). The assessment of baroreflex gain by means of the sequence method (total gain, up sequence gain, and down sequence gain) also revealed an improvement in baroreflex function in the 2K1C + EA group compared to the 2K1C group (Figures 2(c)–2(e)).

### 3.2. Effect of EA Treatment on GABA_B_ Receptor-Mediated Responses in NTS

**Figure 3:**
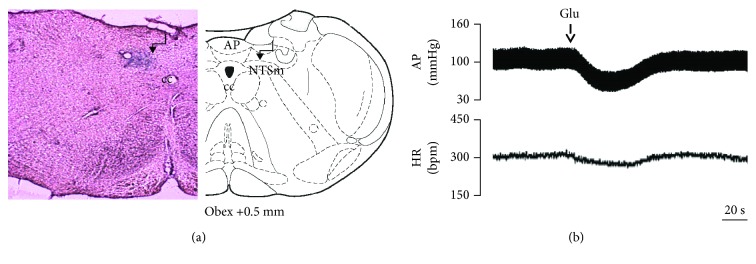
Identification of the microinjection sites in the nucleus tractus solitarii (NTS). (a) A representative photomicrograph showing the NTS microinjection site and the location of this microinjection site based on the rat brain atlas. The arrow indicates the injection site. (b) Functional identification of the NTS with microinjections of glutamate (Glu; 300 pmol in 50 nL) in one rat. Unilateral glutamate produced decreases in arterial pressure (AP) and HR.

**Figure 4:**
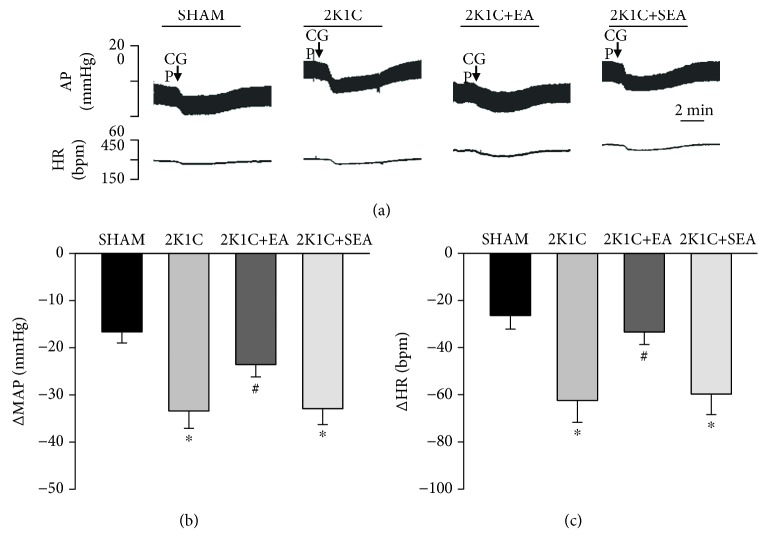
Effect of the GABA_B_ receptor antagonist, CGP-35348, microinjected into the NTS on MAP and HR in the sham, 2K1C, 2K1C + EA, and 2K1C + SEA groups. (a) Representative original tracings showing AP and HR changes evoked by microinjection of CGP-35348 (100 pmol in 50 nL) into the NTS in the sham, 2K1C, 2K1C + EA, and 2K1C + SEA groups. The horizontal bar represents recording duration of 2 min. Arrows indicates the injection of CGP-35348 (CGP). The peak alteration (Δ) of MAP (b) and peak alteration (Δ) of HR (c) after microinjection of CGP-35348 (100 pmol in 50 nL) into the NTS of the sham, 2K1C, 2K1C + EA, and 2K1C + SEA groups. Values are means ± SE (*n* = 7–8 in each group). ^∗^*P* < 0.05 vs. sham group; #*P* < 0.05 vs. 2K1C group.

To investigate the effect of EA treatment on GABA_B_ receptor function in the NTS, GABA_B_ receptor antagonist CGP-35348 or GABA_B_ receptor agonist baclofen was microinjected into the NTS of rats. As shown in Figure 3, the microinjection sites in the NTS were identified. Bilateral microinjection of CGP-35348 (100 pmol, 50 nL) into the NTS decreased MAP and HR in varying degrees in different groups (Figure 4(a)). The response to CGP-35348 microinjected into the NTS was greater in the 2K1C group when compared with that in the sham group. Treatment with EA for 14 days normalized the response to CGP-35348 in 2K1C hypertensive rats (Figure 4(b)). No differences in MAP change were found between the 2K1C + SEA group and the 2K1C group. Changes in HR after microinjection of CGP-35348 into the NTS followed the same pattern as MAP (Figure 4(c)).

**Figure 5:**
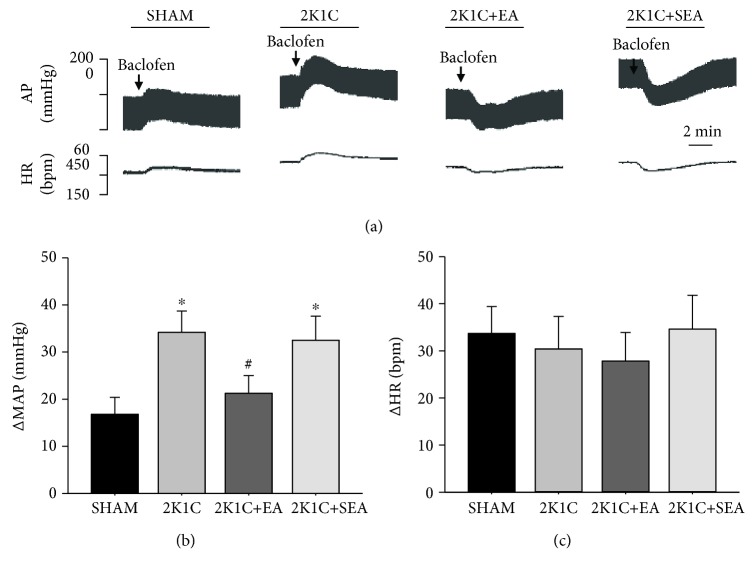
Effect of the GABA_B_ receptor agonist baclofen on MAP and HR in the sham, 2K1C, 2K1C + EA, and 2K1C + SEA groups. (a) Representative original tracings showing AP and HR changes evoked by microinjection of baclofen (50 pmol in 50 nL) into the NTS in the sham, 2K1C, 2K1C + EA, and 2K1C + SEA groups. The peak alteration (Δ) of MAP (b) and peak alteration (Δ) of HR (c) after microinjection of baclofen (50 pmol in 50 nL) into the NTS. Values are means ± SE (*n* = 7–8 in each group). ^∗^*P* < 0.05 vs. sham group; #*P* < 0.05 vs. 2K1C group.

As shown in Figures 5(a) and 5(b), microinjection of baclofen into the NTS induced an elevation in BP in varying degrees in different groups. The amplitudes of MAP change induced by baclofen in the 2K1C group were significantly greater than those in the sham group. In contrast, treatment with EA for 14 days significantly reduced the MAP change in the 2K1C + SEA group when compared with the 2K1C group. Changes in HR after microinjection of baclofen into NTS followed the same pattern as MAP (Figure 5(c)).

### 3.3. Effect of EA Treatment on GABA_A_ Receptor-Mediated Responses in NTS

**Figure 6:**
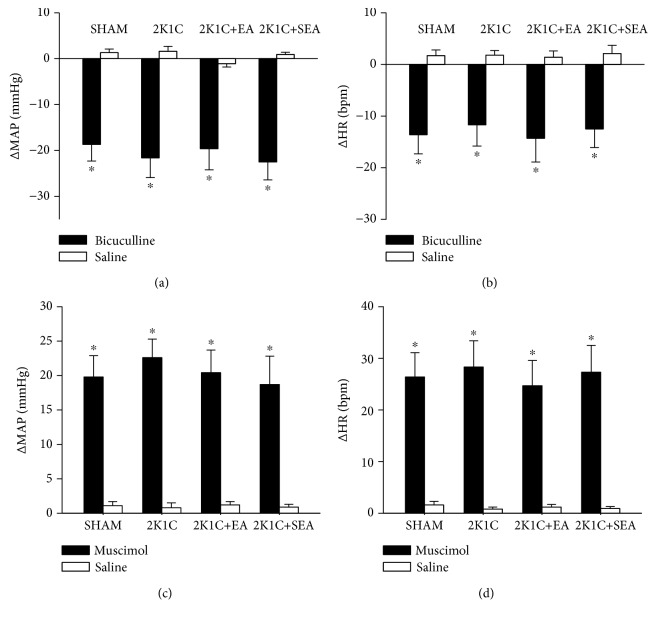
Effect of the GABA_A_ receptor antagonist bicuculline and agonist muscimol on MAP and HR in the sham, 2K1C, 2K1C + EA, and 2K1C + SEA rats. MAP (a) and HR (b) changes evoked by microinjection of bicuculline (10 pmol in 50 nL) into the NTS. MAP (c) and HR (d) changes evoked by microinjection of muscimol (100 pmol in 50 nL) into the NTS. Values are means ± SE (*n* = 7 or 5 rats in each group). ^∗^*P* < 0.05 vs. saline control in each group.

To investigate the effect of EA treatment on GABA_A_ receptor function in the NTS, the bicuculline or muscimol was microinjected into the NTS of rats. Microinjection of bicuculline (10 pmol in 50 nL) into the NTS decreased MAP and HR in all investigated groups. As shown in Figures 6(a) and 6(b), there were no significant differences in peak MAP and HR changes after bicuculline microinjection among the sham, 2K1C, 2K1C + EA, and 2K1C + SEA groups. In addition, microinjection of muscimol (100 pmol in 50 nL) into the NTS significantly increased MAP and HR in rats of all four groups. As shown in Figures 6(c) and 6(d), similar to bicuculline response, the peak MAP and HR changes after muscimol microinjection exhibited no significant differences among the sham, 2K1C, 2K1C + EA, and 2K1C + SEA groups.

### 3.4. Effect of EA Treatment on GABA_B_ and GABA_A_ Receptor Expressions in NTS

**Figure 7:**
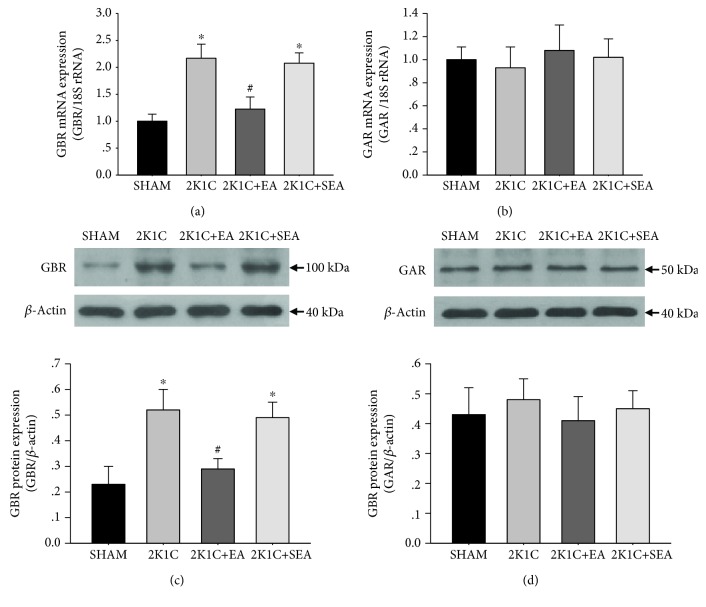
Effect of long-term EA treatment on GABA_B_ and GABA_A_ receptor expressions in the NTS of rats. (a, b) GABA_B_ receptor (GBR) and GABA_A_ receptor (GAR) mRNA levels within the NTS in the sham, 2K1C, 2K1C + EA, and 2K1C + SEA groups which were detected with real-time RT-PCR. Data were normalized with 18 S rRNA. (c, d) Quantitative analysis of GABA_B_ receptor (GBR) and GABA_A_ receptor (GAR) protein levels within the NTS in the sham, 2K1C, 2K1C + EA, and 2K1C + SEA groups. The upper panel shows the representative immunoblots of GABA_B_ receptor (GBR) and GABA_A_ receptor (GAR) protein levels within the NTS in each group. Values are normalized using *β*-actin. Values are means ± SE (*n* = 6 in each group). ^∗^*P* < 0.05 vs. the sham group; ^#^*P* < 0.05 vs. the 2K1C group.

GABA_B_ and GABA_A_ receptor mRNA levels in the NTS were detected with real-time RT-PCR. The results shown in Figure 7(a) demonstrate that GABA_B_ receptor mRNA levels in the NTS increased in the 2K1C group, and EA attenuated this change. There were no differences in the levels of GABA_A_ receptor mRNA among groups. In addition, GABA_B_ and GABA_A_ receptor protein levels were determined by Western blot analysis in the micropunched NTS in rats. Data in Figure 7(c) demonstrate that the 2K1C group presented a twofold increase in GABA_B_ expression levels compared with the sham group, and EA treatment significantly reduced GABA_B_ expression in the 2K1C + EA group when compared to the 2K1C group. In contrast, the expression of GABA_A_ receptor was comparable between the sham, 2K1C, 2K1C + EA, and 2K1C + SEA groups (Figures 7(b) and 7(d)).

## 4. Discussion

The main findings of our present study demonstrated that (1) long-term EA treatment at ST36 lowers the increased blood pressure and improves the depressed baroreflex response in 2K1C hypertensive rats, (2) the enhanced responses to a GABA_B_ receptor antagonist or an agonist microinjected into the NTS of hypertensive rats were significantly reversed by long-term EA treatment at ST36, and (3) EA treatment reduced the increased GABA_B_ receptor expression in the NTS of the hypertensive rat. These results suggest that the BP-lowering action of EA treatment is related to the modulation of functional GABA_B_ receptor expression and inhibitory GABAergic neurotransmission in the NTS.

Both clinical and animal studies indicate that acupuncture at certain acupoints is capable of reducing arterial BP and ameliorating end-organ damage in hypertension and has been recommended as an effective nonpharmacological treatment [1, 2]. It has been demonstrated that EA at the ST36 acupoint is most effective in treating cardiovascular diseases. For example, stimulation of the ST36 acupoint overlying the deep peroneal nerves inhibits sympathoexcitatory reflex responses induced by gastric distension and gall bladder stimulation [12, 28, 29]. Therefore, the ST36 point was chosen in the present study to investigate the long-term effect of EA on 2K1C hypertensive rats, and we demonstrated that two-week acupuncture significantly decreased high BP in 2K1C rats.

The baroreflex is one of the most powerful and rapidly acting mechanisms for controlling arterial BP. It has been suggested that arterial baroreflex is involved in the long-term control of BP [30]. Several studies have shown that the sensitivity of the baroreflex is diminished in several forms of hypertension [22, 31, 32]. In addition, patients with baroreflex failure suffer from a volatile hypertension with sympathetic nerve activity increased for years [33]. The results from the present study supported the insights that 2K1C hypertensive rats exhibit the reduced baroreflex sensitivity and that treatment with EA at the ST36 point could restore this sensitivity. Similar results have been reported for aortic-denervated rabbits and heart failure rats [10, 34, 35], supporting the notion that EA-induced changes in baroreceptor activity may be involved in blunted sympathoexcitation responses.

The NTS is the primary termination site for a variety of cardiovascular afferents. Incremental evidence indicates that the NTS is involved in mediating the central interaction between baroreceptor and somatosensory receptor inputs [13, 14, 36, 37]. This information is integrated and via projections to the rostral and caudal ventrolateral medulla or to the ambiguous nucleus, and then the reflex control of autonomic nerve activity occurs. It has been demonstrated that sensory stimulation of the hindlimb somatic afferent modifies neuronal activities in the NTS [13], and resection of the deep peroneal nerve eliminates the therapeutic effect of EA on the ST36 acupoint [29, 35, 38]. However, the precise route by which sensory signals elicited from acupuncture transits the NTS to activate outgoing regulation signals needs further investigation. GABA appears to be the primary inhibitory neurotransmitter in the NTS, and enhancement of GABAergic neurotransmission in the NTS tends to increase sympathetic outflow and has been implicated in various forms of experimental hypertension [15, 16]. In the present study, we confirmed that 2K1C rats had enhanced responses of GABA_B_ receptors in the NTS and that EA significantly decreased the function of GABA_B_ receptors in the NTS in renovascular hypertensive rats. These results suggest that EA is capable of attenuating the increased GABAergic inhibition in the NTS, leading to restoring baroreflex central gain and sensitivity. However, the detailed mechanisms of EA on GABA_B_ receptor function in the NTS of renovascular hypertensive rats have not been firmly established.

The GABA_B_ receptor is a G protein-coupled receptor that mediates slow and prolonged inhibitory neurotransmission in the brain [15]. The pressor response to baclofen microinjected into the NTS is elevated in spontaneously hypertensive rats, deoxycorticosterone acetate salt-induced hypertensive rats, renal wrap hypertensive rats, and angiotensin II infusion-induced hypertensive rats [18–21]. Enhanced expression of the GABA_B_ receptor in the NTS has also been reported in hypertensive rat models, such as Ang II-induced hypertensive rats and renal wrap hypertensive rats [18, 21, 39]. Consistent with these observations, the present study results indicated that the GABA_B_ receptor, but not GABA_A_ expression, was increased in the NTS of 2K1C hypertensive rats. However, little is known about the cellular mechanisms of enhanced GABA_B_ receptor expression in the NTS of hypertensive rats. One possibility is that enhanced GABA_B_ receptor expression in the NTS is caused by high BP, because elevated peripheral arterial pressure may increase the central input signal, leading to altered gene expression in brain cardiovascular regulatory areas. This seems unlikely, however, because we have previously observed that GABA_B_ receptor expression is unaltered in the NTS of rats made hypertensive by peripheral infusion of N (*ω*)-nitro-L-arginine methyl ester [21]. A second possibility is that enhanced GABA_B_ receptor expression in 2K1C rats is caused by angiotensin II, because angiotensin II activity in the NTS is increased in 2K1C rats, and angiotensin II increases GABA_B_ expression [21, 40, 41]. Therefore, identifying the exact signaling pathways controlling gene regulation that are altered and lead to elevated GABA_B_ receptor expression in the NTS requires further investigation.

In summary, our data demonstrate that renovascular hypertension alters the function and expression of the GABA_B_ receptor in the NTS and increases GABAergic inhibition in the NTS, which partly leads to an increase in MAP and sympathetic activity. This indicates that EA attenuates hypertension partly through the GABA_B_ receptor pathway in renovascular hypertensive rats. Our findings provide further evidence and insight into the beneficial effect of EA on renovascular hypertension.

## Data Availability

The datasets generated during and/or analyzed during the current study are available from the corresponding author on reasonable request.
